# An Intuitionistic Evidential Method for Weight Determination in FMEA Based on Belief Entropy

**DOI:** 10.3390/e21020211

**Published:** 2019-02-22

**Authors:** Zeyi Liu, Fuyuan Xiao

**Affiliations:** School of Computer and Information Science, Southwest University, Chongqing 400715, China

**Keywords:** failure mode and effects analysis, evidence theory, belief entropy, intuitionistic fuzzy set, evidence distance, weight

## Abstract

Failure Mode and Effects Analysis (FMEA) has been regarded as an effective analysis approach to identify and rank the potential failure modes in many applications. However, how to determine the weights of team members appropriately, with the impact factor of domain experts’ uncertainty in decision-making of FMEA, is still an open issue. In this paper, a new method to determine the weights of team members, which combines evidence theory, intuitionistic fuzzy sets (IFSs) and belief entropy, is proposed to analyze the failure modes. One of the advantages of the presented model is that the uncertainty of experts in the decision-making process is taken into consideration. The proposed method is data driven with objective and reasonable properties, which considers the risk of weights more completely. A numerical example is shown to illustrate the feasibility and availability of the proposed method.

## 1. Introduction

Failure Mode and Effects Analysis (FMEA) has received attention from many researchers [1,2,3,4,5,6,7], and it can evaluate and analyze various risks in order to reduce these risks to acceptable levels or directly eliminate them. Moreover, FMEA is a very complex system so that information fusion technology is used in evaluation processes, such as evidence theory [8,9] and D number [10]. Since the uncertainty information is inevitable in FMEA, some methods have been widely used, such as Dempster–Shafer evidence theory and so on [11,12,13].

Though FMEA has been used in practice for many years, how to determine the weights of risk factors and team members is still an open issue. In order to define the weights more reasonably, some scholars have proposed many methods. The intuitionistic fuzzy entropy is introduced by Lei and Wang [14] to determine the weights of risk factors, while, in [15], the weights of risk factors are calculated by the objective weights. While Boran et al. [16] determined the subjective weights of risk factors. In the method proposed in [17], the weights of risk factors are simply determined by the weights calculation proposed by Boran et al. [16], although the intuitionistic fuzzy set (IFS) model is efficient to deal with FMEA [18]. However, existing methods do not take the uncertainty into consideration for the relative importance of team members.

In recent years, the relative concept of intelligence has been paid great attention due to the simulation of human intelligence [19,20]. As a result, it is reasonable to model experts’ uncertainty in the process of decision-making in FMEA, which is important to improve the intelligent degree of the evaluation system. Thus, the measurement of uncertainty should also be regarded as content worth exploring. The related research of uncertainty metrics has been heavily discussed [21,22,23,24]. For probability distributions, Shannon entropy is efficient to handle the uncertainty [25]. However, it can not deal with the uncertainty of basic probability assignment (BPA) in Dempster–Shafer evidence theory [26]. To address this issue, a new belief entropy, named Deng entropy, is presented [26]. In recent years, the belief entropy has been widely used in many fields [27,28].

In this paper, a hybrid weights determination of team members in the FMEA model is proposed based on the evidence distance [29] and the belief entropy [26]. The evidence distance is to measure the degree of conflict for all team members, and the belief entropy is used to model the domain experts’ uncertainty in FMEA. With the combination of evidence distance, the new weights of team members are obtained, which makes the final rank of failure modes be more effective and reasonable.

The rest of this paper is organized as follows. In Section 2, some basic definitions about the evidence theory, IFS, and belief entropy are briefly introduced. In Section 3, the new method to determine the weights of team members is proposed. In Section 4, a numerical example and the computational process are illustrated. Furthermore, the comparisons and discussion have been also mentioned. In Section 5, some conclusions of the proposed method are drawn.

## 2. Preliminaries

In this section, some basic concepts which include evidence theory, intuitionistic fuzzy sets and belief entropy will be introduced.

### 2.1. Evidence Theory

Evidence theory was developed by Shafer and was firstly proposed by Dempster [30], thus it is also called D–S evidence theory. Recently, theoretical research on evidence theory has played a very important role in many applications such as decision-making [31,32,33,34], complex networks [35,36], fault diagnosis [37,38,39,40,41], classification [42,43] and so on [44,45,46,47].

In evidence theory [30], there is a fixed set of *N* mutually exclusive and exhaustive elements, called the frame of discernment, which is symbolized by $\Omega =\{H_{1},H_{2},H_{3}\cdots H_{N}\}$. P(Ω) is denoted as the power set composed of $2^{N}$ elements of Ω. Each element of $2^{N}$ represents a proposition [48].

**Definition** **1.** *A basic probability assignment (BPA) is a function. The range is from P(Ω) to [0,1], which is defined by [30]*

(1) m:P(Ω)→[0,1],A↦m(A)

*and it must satisfy the following conditions:*

(2) $$\sum_{A\in P(\Omega )}m(A)=1,m\left(\emptyset \right)=0.$$

*The mass m(A) indicates the strength of the evidence’s support for A, while m(Ω) is represented as the uncertainty of evidence. If m(Ω) = 1, no useful information from the evidence exists.*

The basic probability assignment (BPA) function, plausibility function (PF), belief function (BF) and other trust quantization functions are described as follows. Each function has a clear definition with physical meaning and there are some corresponding relationships.

**Definition** **2.** *Given a BPA m, for a proposition A⊆*Ω*, the belief function Bel: $2^{\Omega }$→ [0,1] is defined as [30]*

(3) $$Bel\left(A\right)=\sum_{B\subseteq A}m\left(B\right).$$

*The plausibility function Pl: $2^{\Omega }$→ [0,1] is defined as*

(4) $$Pl\left(A\right)=1-Bel\left(\bar{A}\right)=\sum_{B⋂A\neq \emptyset }m\left(B\right),$$

*where $\bar{A}$ = *Ω*−A. The quantity of Bel(A) can be seen as a measure of people’s belief that the hypothesis A is true and can be viewed as a lower limit function on the probability of A. The plausibility Pl(A) can be interpreted as the degree that we absolutely believe in A and can be seen as an upper limit function on the probability of A.*

Based on the classical evidence theory, the combination rules to aggregate the multiple sources are defined as follows.

**Definition** **3.** *Assume that there are two bodies of evidence $m_{1}$ and $m_{2}$ defined on *Ω*, respectively, $m_{1}$ and $m_{2}$ can be combined with Dempster’s orthogonal rule as follows [48,49]:*

(5) $$m_{1}⊕m_{2}=m\left(A\right)=\frac{\sum_{B⋂C=A}m_{1}\left(B\right)m_{2}\left(C\right)}{1-K},$$

*where*

(6) $$K=\sum_{B⋂C=\emptyset }m_{1}\left(B\right)m_{2}\left(C\right),$$

*where K (conflict coefficient) is called the degree of conflict which measures the degree of conflict between $m_{1}$ and $m_{2}$. If K=0, it means that there is no conflict between $m_{1}$ and $m_{2}$, and if K=1, it means that $m_{1}$ and $m_{2}$ is a complete contradiction. In recent years, more and more scholars have paid attention to improve the method of combination rules [50,51].*

### 2.2. Evidence Distance

Evidence distance is regarded as an effective method to measure the conflict of evidence. Here are some of the basic concepts:

**Definition** **4.** *Assume that $m_{1}$ and $m_{2}$ are two BPAs defined on the same frame of discernment *Ω*, which contains N mutually exclusive and exhaustive hypotheses. Namely, it can be expressed as $\Omega =\{H_{1},H_{2},\ldots ,H_{N}\}$. The basic definition can be defined as follows [29]:*

(7) $$d_{BPA}\left(m_{1},m{}_{2}\right)=\sqrt{\frac{1}{2}{\left(\vec{m_{1}}-\vec{m_{2}}\right)}^{T}\underset{=}{D}\left(\vec{m_{1}}-\vec{m_{2}}\right)},$$

*where $\vec{m_{1}}$ and $\vec{m_{2}}$ are the BPAs and $\underset{=}{D}$ is an $2^{N}\times 2^{N}$ matrix where the elements are $D\left(A,B\right)=\frac{\left|A\cap B\right|}{\left|A\cup B\right|}$, and A,B⊆U. Another way to represent $d_{BPA}$ is*

(8) $$d_{BPA}\left(m_{1},m_{2}\right)=\sqrt{\frac{1}{2}\left(\right\|\vec{m_{1}}{\|}^{2}+\|\vec{m_{2}}{\|}^{2}-2<\vec{m_{1}},\vec{m_{2}}>)},$$

*where $\|\vec{m_{1}}{\|}^{2}\;=\;<\vec{m},\vec{m}>$, and $<\vec{m_{1}},\vec{m_{2}}>$ is the scalar product defined by*

(9) $$<\vec{m_{1}},\vec{m_{2}}>=\sum_{i=1}^{2^{n}}\sum_{j=1}^{2^{n}}m_{1}\left(A_{i}\right)m_{2}\left(A_{j}\right)\frac{|A_{i}\cap A_{j}|}{|A_{i}\cup A_{j}|}$$

*with $A_{i},A_{j}\in P\left(\Omega \right)$, i,j = 1,2,..., $2^{N}$.*

To combine the multiple source of evaluation and better solve the combination issues of highly conflicting evidence, the weighted average method is proposed by Deng et al. [52].

### 2.3. Intuitionistic Fuzzy Set

Since the intuitionistic fuzzy set (IFS) is proposed by Atanassov as a generalization of fuzzy sets in 1986, the aggregation of fuzzy sets and IFS theory have received a lot of attention in the past few years [53,54]. In a classical fuzzy sets theory, the relationship between each set is only Belongto or NotBelong to. The central idea of the traditional fuzzy set is to expand the characteristic function to the closed interval $\left[0,1\right]$. Based on it, the intuitionistic fuzzy sets were introduced to express the uncertain information better. Here are some of the basic definitions:

**Definition** **5.** *An intuitionistic fuzzy set (IFS) A on the space X is defined by two functions, $A=<A^{+},A^{-}>$, $A^{+}\left(x\right)$ can be represented by the degree of membership of x in A and $A^{-}\left(x\right)$ can be represented by the degree of nonmembership of x in A. Furthermore, it satisfies the condition that [55]*

(10) $$0\leq A^{+}\left(x\right)+A^{-}\left(x\right)\leq 1,$$

*where $A^{+}\left(x\right)\in \left[0,1\right]$ and $A^{-}\left(x\right)\in \left[0,1\right]$.*

*The degree of hesitancy of x is defined as*

(11) $$Hes\left(x\right)=1-(A^{+}\left(x\right)+A^{-}\left(x\right)).$$

*Thus, the membership grade of x in the IFS A can be expressed by the tuple $A\left(x\right)=<A^{+}\left(x\right),A^{-}\left(x\right)>$.*

With the development of IFS and evidence, the relationship between these two mathematical models has been investigated more and more. Here is a brief introduction.

Assume that there exists an IFS $A=\{<x,\mu_{A}\left(x\right),\nu_{A}\left(x\right)>|x\in X\}$. The three kinds of variables are differentiated and denoted x∈A, x∉A, and the situation of hesitation when both the two hypotheses can not be approved or rejected. In this case above, the relationship between IFS and evidence theory by mathematical modelling can be found, which can be expressed that [56]

(12) $$m\left(Yes\right)=\mu_{A}\left(x\right),$$

(13) $$m\left(No\right)=\nu_{A}\left(x\right),$$

(14) $$m\left(Yes,No\right)=\pi_{A}\left(x\right).$$

Recalling the evidence theory, the IFS *A* can also be expressed as another form [56]

(15) $$A=\{<x,BI_{A}\left(x\right)>|x\in X\},$$

where

(16) $$Bel_{A}\left(x\right)=m\left(Yes\right)=\mu_{A}\left(x\right),$$

(17) $$\begin{array}{c} Pl_{A}\left(x\right)=m\left(Yes\right)+m\left(Yes,No\right)\\ =\mu_{A}\left(x\right)+\pi_{A}\left(x\right)\\ =1-\upsilon_{A}\left(x\right). \end{array}$$

Thus, the belief interval of proposition *A* is defined as follows:

(18) $$BI_{A}\left(x\right)=\left[Bel_{A}\left(x\right),Pl_{A}\left(x\right)\right].$$

**Definition** **6.** *Assume that there exist two alternatives $x_{i}$ and $x_{j}$. Based on the conversion process, the belief interval for those two alternatives can be defined as follows [57]:*

(19) $$\begin{array}{c} P\left(x_{i}>x_{j}\right)=\frac{\max\left\{0,Pl\left(x_{i}\right)-Bel\left(x_{j}\right)\right\}-\max\left\{0,Bel\left(x_{i}\right)-\mathrm{Pl}\left(x_{j}\right)\right\}}{\left[Pl\left(x_{i}\right)-Bel\left(x_{i}\right)\right]+\left[Pl\left(x_{j}\right)-Bel\left(x_{j}\right)\right]}, \end{array}$$

*where $P(x_{i}>x_{j})$ expressed the degree of possibility of $x_{i}$>$x_{j}$.*

### 2.4. Belief Entropy

In the classical information science, Shannon entropy has been used in many applications [58]. Here are some of the basic definitions:

**Definition** **7.** *Shannon entropy is defined as [25]*

(20) $$H=-\sum_{i=1}^{N}p_{i}\log_{b}p_{i},$$

*where N is the number of basic states, $p_{i}$ denotes the probability of state i, and $p_{i}$ satisfies*

(21) $$\sum_{i=1}^{N}p_{i}=1.$$

*If the unit information is bit, then b=2, Shannon entropy is expressed as*

(22) $$H=-\sum_{i=1}^{N}p_{i}\log_{2}p_{i}.$$

However, since Dempster–Shafer evidence theory has been widely used in many fields, the method to measure the uncertainty in evidence theory is still an issue worth exploring. To measure the uncertain information better, a belief entropy, named as Deng entropy, is presented to deal with uncertainty measure of BPA based on Shannon entropy [25]. Here are some of the basic definitions:

**Definition** **8.** *In frame of discernment X, the belief entropy is defined as [26]*

(23) $$E_{d}\left(m\right)=-\sum_{A\subseteq X}m\left(A\right)\log_{2}\frac{m(A)}{2^{\left|A\right|}-1},$$

*where $\left|A\right|$ is the cardinality of the proposition A and $E_{d}\left(m\right)$ expresses the belief entropy for basic possibility assignments. In particular, the belief entropy can definitely degenerate to the Shannon entropy if the belief is only assigned to single elements. With the development of evidence theory, the belief entropy has been more and more researched [59,60].*

## 3. The Proposed Method

In this section, a new method to determine the weights of team members based on the evidence theory, intuitionistic fuzzy sets and belief entropy is proposed to rank the failure modes. The function of Failure Mode and Effects Analysis (FMEA) team members is to assess the risk factors with linguistic variables, such as very low, low, medium, high, very high and so on. Assume that there are *k* cross-functional team members $TM_{k}\left(k=1,\ldots ,p\right)$ in an FMEA team; after discussing them, the experts prioritize *i* potential failure modes $FM_{i}\left(i=1,\ldots ,m\right)$. Each failure mode is evaluated on the *j* risk factor $RF_{i}\left(j=1,2,3\right)$. $\lambda_{ij}^{k}$ is the weight of decision makers which reflects the relative importance of the *k*th decision maker with respect to the *j*th risk factors for the *i*th potential failure modes. In addition, intuitionistic fuzzy numbers [61], which are represented by the ordered pairs of membership degrees and non-memberships corresponding to the intuitionistic fuzzy sets, is used to simply express the relevant conversion process.

**Figure 1:**
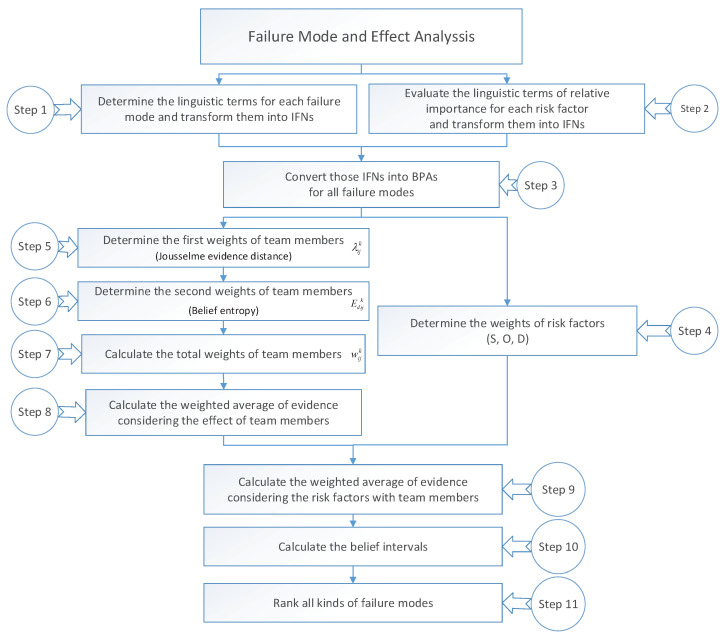
The flow chart of our proposed method.

Assume that the IFN $\alpha_{ij}^{k}=\left(\mu_{ij}^{k},\upsilon_{ij}^{k}\right)$ is provided by $TM_{k}$ on the assessment of $FM_{i}$ for $RF_{j}$. The proposed method consists of eleven steps. In addition, the flowchart of the proposed approach is shown in Figure 1.

Step 1: Determine the linguistic terms for each failure mode and transform them into IFNs. The specific judgement levels are divided into ten linguistic parts (see in Table 1, Table 2 and Table 3), which contains Very very low (VVL), Very Low (VL), Low (L), Medium low (ML), Medium (M), Medium high (MH), High (H), Very high (VH), Very very high (VVH) and Extremely high (EH).

Step 2: Evaluate the linguistic terms of relative importance for each risk factor and transform them into IFNs. Similarly, the specific judgement levels are divided into five parts (see in Table 4), which contains Very important, Important, Medium, Unimportant and Very unimportant.

Step 3: Convert all IFNs into BPAs for all failure modes. In addition, the concrete form can be defined as follows:

(24) $$m_{ij}^{k}\left(Yes\right)=\mu_{ij}^{k},$$

(25) $$m_{ij}^{k}\left(No\right)=\upsilon_{ij}^{k},$$

(26) $$m_{ij}^{k}\left(Yes,No\right)=1-\mu_{ij}^{k}-\upsilon_{ij}^{k}.$$

Step 4: Determine the weights of risk factors. For the three judgement model S, O and D, each model can be transformed into an IFS to represent the information value. Based on the weight calculation, which is proposed by Boran et al. [16], the weight $w_{j}$ can be obtained. The computational equations are defined as follows:

(27) $$w_{j}=\frac{\left(\mu_{j}+\pi_{j}\left(\mu_{j}/\left(\mu_{j}+\upsilon_{j}\right)\right)\right)}{\sum_{j=1}^{n}\left(\mu_{j}+\pi_{j}\left(\mu_{j}/\left(\mu_{j}+\upsilon_{j}\right)\right)\right)},$$

which satisfy the condition that

(28) $$\sum_{j=1}^{n}w_{j}=1.$$

Step 5: Determine the weights of team members $\lambda_{ij}^{k}$ by using evidence distance which is introduced by Jousselme et al. [29].

Assume two groups of BPAs $m_{ij}^{q}\left(Yes\right),m_{ij}^{q}\left(No\right),m_{ij}^{q}\left(Yes,No\right)$ and $m_{ij}^{t}\left(Yes\right),m_{ij}^{t}\left(No\right),m_{ij}^{t}\left(Yes,No\right)$, (q,t=1,2,…,p) are two bodies of evidence (BOE), obtained by two different team members. In this paper, the similarity function, using the evidence distance to define the distance $d(m_{ij}^{q},m_{ij}^{t})$ between $m_{ij}^{q}$ and $m_{ij}^{t}$, is proposed as follows:

(29) $$s\left(m_{ij}^{q},m_{ij}^{t}\right)=1-d\left(m_{ij}^{q},m_{ij}^{t}\right).$$

The $Sup(m_{ij}^{k})$ is used to represent the degree of $m_{ij}^{k}$ supported by other bodies of evidence. In addition, the reliability degree $Crd(m_{ij}^{k})$ are defined as follows:

(30) $$Crd\left(m_{ij}^{k}\right)=\frac{Sup(m_{ij}^{k})}{\sum_{t=1}^{p}Sup(m_{ij}^{t})},$$

(31) $$Sup\left(m_{ij}^{k}\right)=\sum_{t=1,t\neq k}^{p}s(m_{ij}^{k},m_{ij}^{t}).$$

The $Crd(m_{ij}^{k})$ is to define the $\lambda_{ij}^{k}$

(32) $$\lambda_{ij}^{k}=Crd\left(m_{ij}^{k}\right)=\frac{Sup(m_{ij}^{k})}{\sum_{t=1}^{p}Sup(m_{ij}^{t})}.$$

Step 6: Determine the weights of team members ${E_{d}}_{ij}^{k}$ by using belief entropy [26]. Based on the fellow steps, for all team members, their information value has been transformed into IFSs. Then, another weight ${E_{d}}_{ij}^{k}$ is calculated, which expresses the amount of uncertainty for all propositions. The specific equation is defined as follows:

(33) $$\begin{array}{c} {E_{d}}_{ij}^{k}=\\ -m_{ij}^{k}\left(Y\right)\log_{2}\frac{m_{ij}^{k}\left(Y\right)}{2^{\left|Y\right|}-1}-m_{ij}^{k}\left(N\right)\log_{2}\frac{m_{ij}^{k}\left(N\right)}{2^{\left|N\right|}-1}-m_{ij}^{k}\left(Y,N\right)\log_{2}\frac{m_{ij}^{k}\left(Y,N\right)}{2^{\left|Y,N\right|}-1}. \end{array}$$

Step 7: Calculate the total weights of team members $w_{ij}^{k}$ by combing $\lambda_{ij}^{k}$ and ${E_{d}}_{ij}^{k}$. After obtaining the two weights, the total weights of FMEA team members can be calculated as the form of multiplication, which is defined as follows:

(34) $$w_{ij}^{k}=\frac{\lambda_{ij}^{k}{E_{d}}_{ij}^{k}}{\sum_{n=1}^{p}\lambda_{ij}^{k}{E_{d}}_{ij}^{k}},$$

(35) $$\sum_{k=1}^{p}w_{ij}^{k}=1,$$

where *p* is the total number of failure modes for each risk factor.

Step 8: Calculate the weighted average of evidence considering the team members’ effect of FMEA model. For each failure mode $FM_{i}$, there exists a group of basic probability assignment functions to express the degree of importance, which can be denoted as m(Yes) m(No) and m(Yes,No). Thus, after obtaining the $w_{ij}^{k}$ weights, the weighted average can be obtained as follows:

(36) $$m_{ij}^{\prime \prime }\left(Yes\right)=\sum_{k=1}^{p}w_{ij}^{k}m_{ij}^{k}\left(Yes\right),$$

(37) $$m_{ij}^{\prime \prime }\left(No\right)=\sum_{k=1}^{p}w_{ij}^{k}m_{ij}^{k}\left(No\right),$$

(38) $$m_{ij}^{\prime \prime }\left(Yes,No\right)=1-\sum_{k=1}^{p}w_{ij}^{k}m_{ij}^{k}\left(Yes\right)-\sum_{k=1}^{p}w_{ij}^{k}m_{ij}^{k}\left(No\right).$$

Step 9: Calculate the weighted average of evidence considering the risk factors with team members. To consider the impact of different risk factors (S, O, D), the weighted average of evidence which can be denoted as $m_{i}^{\prime }\left(Yes\right)$, $m_{i}^{\prime }\left(No\right)$ and $m_{i}^{\prime }\left(Yes,No\right)$ is expressed as follows:

(39) $$m_{i}^{\prime }\left(Yes\right)=\sum_{j=1}^{n}w_{j}m_{ij}^{\prime \prime }\left(Yes\right),$$

(40) $$m_{i}^{\prime }\left(No\right)=\sum_{j=1}^{n}w_{j}m_{ij}^{\prime \prime }\left(No\right),$$

(41) $$m_{i}^{\prime }\left(Yes,No\right)=1-\sum_{j=1}^{n}w_{j}m_{ij}^{\prime \prime }\left(Yes\right)-\sum_{j=1}^{n}w_{j}m_{ij}^{\prime \prime }\left(No\right).$$

Step 10: Calculate the belief intervals. After obtaining the weighted average of evidence in Step 9, the belief interval $\left[Bel\left(FM_{i}\right),Pl\left(FM_{i}\right)\right]$ which is used to show the degree of support and opposition can be determined as:

(42) $$Bel\left(FM_{i}\right)=m_{i}^{\prime }\left(Yes\right),$$

(43) $$Pl\left(FM_{i}\right)=m_{i}^{\prime }\left(Yes\right)+m_{i}^{\prime }\left(Yes,No\right).$$

Step 11: Rank all kinds of failure modes. Based on the belief intervals, the risk of different failure model can be compared with others by using Equation (19). After the process of comparison, the list of ranking in FMEA can be obtained.

## 4. Application

In this section, an example is used to illustrate the complete procedures of the proposed method.

The risk evaluation process has a great impact in many fields, such as multi-criteria decision-making (MCDM) [62,63,64,65] and other works [66,67]. In most situations, the weights for each risk factor may change the final result and lead the decision maker to make the undeserved judgement. To modify the process of products production as an easier and lower-cost method, the Failure mode and Effects Analysis play a growing important role in modern society.

Thus, an FMEA team consisting of five functional team members identifies potential failure modes in the electronics manufacturing project and wants to prioritize them in terms of their risk factors such as S (Severity), O (Occurrence) and D (Detection). In addition, twelve failure modes are identified. For the difficulty of evaluating the risk factors, the FMEA team members in this numerical example are supposed to assess them employing the linguistic terms. The specific transforming process is shown in Table 1, Table 2 and Table 3.

Step 1: The assessment information of the twelve failure modes on each risk factor, which was provided by the five team members, can be illustrated in Table 5. Each team member comes from different department, such as manufacturing, engineering, design and technique. Considering their deferent specialities and functions, the weights are determined by their degree of importance.

Step 2: Evaluate the linguistic terms of relative importance for each risk factor and transform them into IFNs (see in Table 6).

Step 3: Convert those IFNs into BPAs for all failure modes. The specific transforming equation is shown in Equations (12)–(14).

Step 4: Determine the weights of risk factors. In this paper, the weights calculation of risk factors in this example is shown in Equations (27) and (28). In addition, the results are shown in Table 7.

Step 5: Determine the first weights of team members $\lambda_{ij}^{k}$ by using evidence distance [29]. The specific value of each mode is shown in Table 8.

Step 6: Determine the second weights of team members ${E_{d}}_{ij}^{k}$ by using belief entropy. In addition, the results are shown in Table 9.

Step 7: Calculate the total weights of team members $w_{ij}^{k}$ by combing $\lambda_{ij}^{k}$ and ${E_{d}}_{ij}^{k}$. After the process of normalization, the specific value of weights is shown in Table 10.

Step 8: Calculate the weighted average of evidence considering the team members’ effect of FMEA model. The results are shown in Table 11.

Step 9: Calculate the weighted average of evidence considering the risk factors with team members. By introducing the consideration of risk factors, the weighted average of evidence is calculated. In addition, the results are shown in Table 12.

Step 10: Calculate the belief intervals. With the Equations (41) and (42), the final results are also shown in Table 12.

Step 11: Rank all kinds of failure modes. After the process of comparison, the final ranking can be obtained (see in Table 13).

Here, we present some discussion about the proposed method.

In the previous related research, many scholars have tried to enhance the effectiveness and availability based on intuitionistic fuzzy sets, evidence theory and so on. The ranking comparisons of all the related works are shown in Table 13. There are some ranking differences among those methods. In general, the higher ranked models are $FM_{1}$, $FM_{2}$, and the lowest ranked model is $FM_{12}$, which is consistent with the previous three methods. For other failure modes, it can be seen that, in the evaluation of the proposed method, the overall ordering of $FM_{3}$ and $FM_{4}$ is slightly higher than the previous method, while the remaining rankings are generally consistent. The main reasons are summarized as follows:

The relatively importance of team members are different. In the method proposed by Liu et al. [15], the relative weights were supposed in advance, which are 0.10, 0.15, 0.20, 0.25 and 0.30. In addition, in the intuitionistic fuzzy TOPSIS method, the impacts of team members are not considered. In addition, the method proposed by Guo [17] has just considered the conflict of team members simply. In our proposed method, the weights of team members are defined by using both the evidence distance [29] and the belief entropy [26]. The evidence distance is to show the degree of conflict for all team members. In addition, the belief entropy is used to reflect the uncertainty of the information of each team member. The combination of them can express the evaluated information completely and effectively. To be specific, since the uncertainty information contained in the results of the expert evaluations in $FM_{1}$, $FM_{2}$ and $FM_{12}$ is relatively low in this application, the weights obtained by considering the entropy factor has little effect on the final evaluation. Moreover, in $FM_{3}$ and $FM_{4}$, the overall uncertainty of the expert evaluation is relatively high, the second weights obtained by calculating the belief entropy have a relatively large influence on the overall evaluation result, which leads to the final result as shown in Table 13.

Thus, with the differences mentioned above, the aggregation approaches for all kinds of methods are different. As a comparison, the process of our proposed method to determine the weights of team members is particularly scientific and effective, with strong practical significance and good performance.

## 5. Conclusions

FMEA has been regarded as an effective analysis approach to identify and rank the potential failure modes in many applications. However, the uncertainty of the experts’ decision process is not taken into consideration, which is regarded as an essential factor in decision-making. In this paper, the impact of experts factor uncertainty is modelled. A hybrid method to determine the weights of team members is proposed based on the belief entropy. The application in FMEA illustrates the efficiency of the proposed method.

## Figures and Tables

**Table 1:** The conversion and interpretation of the the relative importance evaluation under risk factor S.

The Linguistic Variables	IFNs	Severity (S)
Very very low (VVL)	(0.10,0.90)	Almost no casualties
Very low (VL)	(0.10,0.75)	Very low level of injuries of people and amount of property damage
Low (L)	(0.25,0.60)	Low level of injuries of people and amount of property damage
Medium low (ML)	(0.40,0.50)	medium level of injuries of people and amount of property damage
Medium (M)	(0.50,0.40)	moderate level of injuries of people and amount of property damage
Medium high (MH)	(0.60,0.30)	moderately high level of injuries of people and amount of property damage
High (H)	(0.70,0.20)	high level of injuries of people and amount of property damage
Very high (VH)	(0.80,0.10)	Very high level of injuries of people and amount of property damage
Very very high (VVH)	(0.90,0.10)	Very very high level of injuries of people and amount of property damage
Extremely high (EH)	(1.00,0.00)	Severe level of injuries or death of people and amount of property damage

**Table 2:** The conversion and interpretation of the the relative importance evaluation under risk factor O.

The Linguistic Variables	IFNs	Occurrence (O)
Very very low (VVL)	(0.10,0.90)	A failure almost unlikely to occur
Very low (VL)	(0.10,0.75)	A failure is likely to occur once, but unlikely to occur more frequently
Low (L)	(0.25,0.60)	A failure occurs in low probabilities
Medium low (ML)	(0.40,0.50)	A failure occurs in moderately low probabilities
Medium (M)	(0.50,0.40)	A failure occurs in moderate probability
Medium high (MH)	(0.60,0.30)	A failure occurs in moderately high probabilities
High (H)	(0.70,0.20)	A failure occurs in high probabilities
Very high (VH)	(0.80,0.10)	A failure occurs in very high probabilities
Very very high (VVH)	(0.90,0.10)	A failure occurs in very very high probabilities
Extremely high (EH)	(1.00,0.00)	A failure occurs in extremely high probabilities

**Table 3:** The conversion and interpretation of the the relative importance evaluation under risk factor D.

The Linguistic Variables	IFNs	Detection (D)
Very very low (VVL)	(0.10,0.90)	The detection of failure occurrence is completely certain
Very low (VL)	(0.10,0.75)	The detection of failure occurrence is almost certain
Low (L)	(0.25,0.60)	The failure occurrence is very likely to be detected
Medium low (ML)	(0.40,0.50)	The failure occurrence is likely to be detected
Medium (M)	(0.50,0.40)	A moderate likelihood to detect the failure occurrence
Medium high (MH)	(0.60,0.30)	A moderately small likelihood to detect the failure occurrence
High (H)	(0.70,0.20)	A small probability of detecting the failure occurrence
Very high (VH)	(0.80,0.10)	A low likelihood to detect the failure occurrence
Very very high (VVH)	(0.90,0.10)	A very low likelihood to detect the failure occurrence
Extremely high (EH)	(1.00,0.00)	Almost impossible to detect failure occurrence

**Table 4:** The linguistic variables for the importance of risk factors.

The Linguistic Variables	IFNs
Very important	(0.90,0.10,0.00)
Important	(0.75,0.20,0.05)
Medium	(0.50,0.45,0.05)
Unimportant	(0.35,0.60,0.05)
Very unimportant	(0.10,0.90,0.00)

**Table 5:** The linguistic evaluation for each failure mode.

Failure Mode	S					O					D				
	${\mathrm{TM}}_{1}$	${\mathrm{TM}}_{2}$	${\mathrm{TM}}_{3}$	${\mathrm{TM}}_{4}$	${\mathrm{TM}}_{5}$	${\mathrm{TM}}_{1}$	${\mathrm{TM}}_{2}$	${\mathrm{TM}}_{3}$	${\mathrm{TM}}_{4}$	${\mathrm{TM}}_{5}$	${\mathrm{TM}}_{1}$	${\mathrm{TM}}_{2}$	${\mathrm{TM}}_{3}$	${\mathrm{TM}}_{4}$	${\mathrm{TM}}_{5}$
$FM_{1}$	VH	VVH	L	H	VH	M	MH	ML	H	MH	ML	ML	MH	M	M
$FM_{2}$	VVH	VH	VH	VVL	VH	M	M	MH	VL	M	ML	MH	MH	H	M
$FM_{3}$	H	MH	VH	L	H	ML	M	L	MH	M	ML	M	L	L	MH
$FM_{4}$	M	ML	H	M	MH	H	M	ML	H	M	ML	ML	H	L	L
$FM_{5}$	L	M	MH	MH	M	H	ML	VVL	ML	L	L	L	ML	ML	M
$FM_{6}$	L	ML	M	L	M	M	MH	L	M	MH	MH	ML	M	ML	ML
$FM_{7}$	M	L	H	M	MH	M	M	L	H	M	ML	M	MH	M	ML
$FM_{8}$	M	ML	MH	L	H	L	MH	MH	L	M	H	MH	L	MH	VL
$FM_{9}$	L	M	H	L	M	M	MH	L	VL	L	L	M	M	ML	L
$FM_{10}$	L	ML	ML	M	M	L	L	L	ML	M	ML	MH	ML	ML	ML
$FM_{11}$	L	M	MH	M	M	L	M	M	M	M	L	M	M	M	M
$FM_{12}$	ML	M	ML	L	MH	M	VL	VL	VL	VL	ML	L	M	VL	L

**Table 6:** The importance of risk factors.

Risk Factors	Mode Abbreviation	The Linguistic Variables
Severity	S	Very important
Occurrence	O	Important
Detection	D	Medium

**Table 7:** The weights of risk factors.

Risk Factors	Weights
Severity	0.4009
Occurrence	0.3516
Detection	0.2475

**Table 8:** The $\lambda_{ij}^{k}$ weights for each failure mode.

Failure Mode	Severity (S)					Occurrence (O)					Detection (D)				
	${\mathrm{TM}}_{1}$	${\mathrm{TM}}_{2}$	${\mathrm{TM}}_{3}$	${\mathrm{TM}}_{4}$	${\mathrm{TM}}_{5}$	${\mathrm{TM}}_{1}$	${\mathrm{TM}}_{2}$	${\mathrm{TM}}_{3}$	${\mathrm{TM}}_{4}$	${\mathrm{TM}}_{5}$	${\mathrm{TM}}_{1}$	${\mathrm{TM}}_{2}$	${\mathrm{TM}}_{3}$	${\mathrm{TM}}_{4}$	${\mathrm{TM}}_{5}$
$FM_{1}$	0.2118	0.2117	0.1463	0.2184	0.2118	0.2035	0.2093	0.1860	0.1919	0.2093	0.2000	0.2000	0.1889	0.2056	0.2056
$FM_{2}$	0.2188	0.2356	0.2356	0.0800	0.2299	0.2157	0.2157	0.1906	0.1625	0.2157	0.1860	0.2093	0.2093	0.1919	0.2035
$FM_{3}$	0.2172	0.2248	0.1939	0.1994	0.1646	0.2044	0.2105	0.1817	0.1929	0.2105	0.2089	0.2032	0.2013	0.2013	0.1851
$FM_{4}$	0.2093	0.1919	0.1860	0.2093	0.2035	0.2000	0.2056	0.1889	0.2000	0.2056	0.2101	0.2101	0.1749	0.2025	0.2025
$FM_{5}$	0.1700	0.2104	0.2046	0.2046	0.2104	0.1638	0.2268	0.1671	0.2268	0.2155	0.1989	0.1989	0.2063	0.2063	0.1896
$FM_{6}$	0.1978	0.2051	0.1997	0.1978	0.1997	0.2104	0.2046	0.1700	0.2104	0.2046	0.1833	0.2056	0.2000	0.2056	0.2056
$FM_{7}$	0.2146	0.1723	0.1900	0.2146	0.2085	0.1889	0.2056	0.2056	0.2000	0.2000	0.1889	0.2056	0.2056	0.2000	0.2000
$FM_{8}$	0.2138	0.2057	0.2075	0.1829	0.1885	0.1940	0.2020	0.2020	0.1940	0.2081	0.1987	0.2199	0.1966	0.2199	0.1648
$FM_{9}$	0.1989	0.2134	0.1753	0.1989	0.2134	0.2008	0.1813	0.2157	0.1864	0.2157	0.1978	0.1997	0.1997	0.2051	0.1978
$FM_{10}$	0.1846	0.2066	0.2066	0.2011	0.2011	0.2099	0.2099	0.2099	0.1689	0.2016	0.2065	0.1739	0.2065	0.2065	0.2065
$FM_{11}$	0.1718	0.2113	0.1941	0.2113	0.2113	0.1697	0.2076	0.2076	0.2076	0.2076	0.2092	0.2092	0.1952	0.2092	0.1772
$FM_{12}$	0.2104	0.2047	0.2104	0.1874	0.1871	0.1466	0.2133	0.2133	0.2133	0.2133	0.2063	0.1989	0.1896	0.2063	0.1989

**Table 9:** The ${E_{d}}_{ij}^{k}$ weights for each failure mode.

Failure Mode	Severity (S)					Occurrence (O)					Detection (D)				
	${\mathrm{TM}}_{1}$	${\mathrm{TM}}_{2}$	${\mathrm{TM}}_{3}$	${\mathrm{TM}}_{4}$	${\mathrm{TM}}_{5}$	${\mathrm{TM}}_{1}$	${\mathrm{TM}}_{2}$	${\mathrm{TM}}_{3}$	${\mathrm{TM}}_{4}$	${\mathrm{TM}}_{5}$	${\mathrm{TM}}_{1}$	${\mathrm{TM}}_{2}$	${\mathrm{TM}}_{3}$	${\mathrm{TM}}_{4}$	${\mathrm{TM}}_{5}$
$FM_{1}$	1.0804	0.4690	1.5904	1.3153	1.0804	1.5195	1.4540	1.5195	1.3153	1.4540	1.5195	1.5195	1.4540	1.5195	1.5195
$FM_{2}$	0.4690	1.0804	1.0804	0.4690	1.3153	1.5195	1.5195	1.4540	1.2918	1.5195	1.5195	1.4540	1.4540	1.3153	1.5195
$FM_{3}$	1.3153	1.4540	1.0804	1.5905	1.3153	1.5195	1.5195	1.5905	1.4540	1.5195	1.5195	1.5195	1.5905	1.5905	1.4540
$FM_{4}$	1.5195	1.5197	1.3153	1.5195	1.4510	1.3153	1.5195	1.5195	1.3153	1.5195	1.5195	1.5195	1.3153	1.5905	1.5905
$FM_{5}$	1.5905	1.5195	1.4510	1.4540	1.5195	1.3153	1.5195	0.4690	1.5195	1.5905	1.5905	1.6483	1.5195	1.5195	1.5195
$FM_{6}$	1.5905	1.5195	1.5195	1.5905	1.5195	1.5195	1.4540	0.9422	1.5195	1.4540	1.4540	1.5195	1.5195	1.5195	1.5195
$FM_{7}$	1.5195	1.5905	1.3153	1.5195	1.4540	1.5195	1.5195	1.5905	1.3153	1.5195	1.5195	1.5195	1.4540	1.5195	1.5195
$FM_{8}$	1.5195	1.5195	1.4510	1.5905	1.3153	1.5905	1.4540	1.4540	1.5905	1.5195	1.3153	1.4540	1.5905	1.4540	1.2918
$FM_{9}$	1.5905	1.5195	1.3153	1.5905	1.5195	1.5195	1.4540	1.5905	1.2918	1.5905	1.5905	1.5195	1.5195	1.5195	1.5905
$FM_{10}$	1.5905	1.5195	1.5195	1.5195	1.5195	1.5905	1.5905	1.5905	1.5195	1.5195	1.5195	1.4540	1.5195	1.5195	1.5195
$FM_{11}$	1.5905	1.5195	1.4540	1.5195	1.5195	1.5905	1.5195	1.5195	1.5195	1.5195	1.5905	1.5905	1.5195	1.5905	1.4540
$FM_{12}$	1.5195	1.5195	1.5195	1.5905	1.4540	1.5195	1.2918	1.2918	1.2918	1.2918	1.5195	1.5905	1.5195	1.2918	1.5905

**Table 10:** The total weights $w_{ij}^{k}$ of team members for each failure mode.

Failure Mode	Severity (S)					Occurrence (O)					Detection (D)				
	${\mathrm{TM}}_{1}$	${\mathrm{TM}}_{2}$	${\mathrm{TM}}_{3}$	${\mathrm{TM}}_{4}$	${\mathrm{TM}}_{5}$	${\mathrm{TM}}_{1}$	${\mathrm{TM}}_{2}$	${\mathrm{TM}}_{3}$	${\mathrm{TM}}_{4}$	${\mathrm{TM}}_{5}$	${\mathrm{TM}}_{1}$	${\mathrm{TM}}_{2}$	${\mathrm{TM}}_{3}$	${\mathrm{TM}}_{4}$	${\mathrm{TM}}_{5}$
$FM_{1}$	0.2125	0.0922	0.2161	0.2667	0.2125	0.2128	0.2095	0.1945	0.1737	0.2095	0.2016	0.2016	0.1822	0.2073	0.2073
$FM_{2}$	0.1078	0.2675	0.2675	0.0394	0.3178	0.2229	0.2229	0.1885	0.1428	0.2229	0.1945	0.2095	0.2095	0.1737	0.2128
$FM_{3}$	0.2107	0.2411	0.1545	0.2339	0.1597	0.2044	0.2105	0.1902	0.1846	0.2014	0.2067	0.2010	0.2085	0.2085	0.1753
$FM_{4}$	0.2166	0.1986	0.1666	0.2166	0.2015	0.1829	0.2172	0.1776	0.1829	0.2173	0.2110	0.2110	0.1521	0.2129	0.2129
$FM_{5}$	0.1797	0.2125	0.1977	0.1977	0.2125	0.1625	0.2599	0.0591	0.2599	0.2585	0.2029	0.2103	0.2010	0.2010	0.1848
$FM_{6}$	0.2033	0.2014	0.1961	0.2033	0.1961	0.2293	0.2133	0.1149	0.2293	0.2133	0.1768	0.2072	0.2016	0.2072	0.2072
$FM_{7}$	0.2204	0.1953	0.1689	0.2204	0.2049	0.1922	0.2092	0.2190	0.1761	0.2035	0.1922	0.2092	0.2190	0.1761	0.2035
$FM_{8}$	0.2198	0.2115	0.2041	0.1968	0.1678	0.2030	0.1934	0.1931	0.2029	0.2079	0.1832	0.2242	0.2192	0.2242	0.1492
$FM_{9}$	0.2093	0.2145	0.1525	0.2093	0.2145	0.2040	0.1762	0.2294	0.1610	0.2294	0.2033	0.1961	0.1961	0.2014	0.2033
$FM_{10}$	0.1916	0.2048	0.2048	0.1993	0.1993	0.2134	0.2134	0.2134	0.1640	0.1958	0.2081	0.1677	0.2081	0.2081	0.2081
$FM_{11}$	0.1799	0.2114	0.1858	0.2114	0.2114	0.1762	0.2059	0.2059	0.2059	0.2059	0.1762	0.2059	0.2059	0.2059	0.2059
$FM_{12}$	0.2103	0.2046	0.2103	0.1960	0.1789	0.1681	0.2080	0.2080	0.2080	0.2080	0.2089	0.2108	0.1920	0.1776	0.2108

**Table 11:** The weighted average of team members.

Failure Mode	Severity (S)			Occurrence (O)			Detection (D)		
	$m_{\mathrm{ij}}^{\prime \prime }\left(\mathrm{Yes}\right)$	$m_{\mathrm{ij}}^{\prime \prime }\left(\mathrm{No}\right)$	$m_{\mathrm{ij}}^{\prime \prime }\left(\mathrm{Yes},\mathrm{No}\right)$	$m_{\mathrm{ij}}^{\prime \prime }\left(\mathrm{Yes}\right)$	$m_{\mathrm{ij}}^{\prime \prime }\left(\mathrm{No}\right)$	$m_{\mathrm{ij}}^{\prime \prime }\left(\mathrm{Yes},\mathrm{No}\right)$	$m_{\mathrm{ij}}^{\prime \prime }\left(\mathrm{Yes}\right)$	$m_{\mathrm{ij}}^{\prime \prime }\left(\mathrm{No}\right)$	$m_{\mathrm{ij}}^{\prime \prime }\left(\mathrm{Yes},\mathrm{No}\right)$
$FM_{1}$	0.6637	0.2347	0.1015	0.5572	0.3428	0.1000	0.4779	0.4221	0.1000
$FM_{2}$	0.7514	0.1633	0.0853	0.4806	0.4123	0.1071	0.5572	0.3428	0.1000
$FM_{3}$	0.5867	0.3022	0.1116	0.4505	0.4400	0.1095	0.3926	0.4865	0.1208
$FM_{4}$	0.5336	0.3663	0.0100	0.5532	0.3467	0.1000	0.3817	0.4970	0.1213
$FM_{5}$	0.4946	0.3964	0.1090	0.3922	0.5007	0.1070	0.3565	0.5018	0.1417
$FM_{6}$	0.3782	0.5014	0.1203	0.5139	0.3803	0.1057	0.4555	0.4444	0.1000
$FM_{7}$	0.5080	0.3828	0.1093	0.4805	0.4086	0.1109	0.4779	0.4221	0.1000
$FM_{8}$	0.4836	0.4065	0.1098	0.4372	0.4425	0.1203	0.4670	0.4146	0.1184
$FM_{9}$	0.4259	0.4532	0.1209	0.3385	0.5305	0.1310	0.3782	0.5014	0.1203
$FM_{10}$	0.4111	0.4793	0.1096	0.3236	0.5444	0.1320	0.4335	0.4665	0.1000
$FM_{11}$	0.4736	0.4714	0.1090	0.4560	0.4352	0.1088	0.4560	0.4352	0.1088
$FM_{12}$	0.4268	0.4634	0.1098	0.1672	0.6911	0.1416	0.3027	0.5673	0.1299

**Table 12:** The weighted average of evidence considering the risk factors with team members and the belief intervals.

Failure Mode	$m_{\mathrm{ij}}^{\prime }\left(\mathrm{Yes}\right)$	$m_{\mathrm{ij}}^{\prime }\left(\mathrm{No}\right)$	$m_{\mathrm{ij}}^{\prime }\left(\mathrm{Yes},\mathrm{No}\right)$	Bel	Pl	Ranking
$FM_{1}$	0.5932	0.3060	0.1007	0.5932	0.6939	2
$FM_{2}$	0.6081	0.2953	0.0966	0.6081	0.7047	1
$FM_{3}$	0.5037	0.3842	0.1121	0.5037	0.6158	4
$FM_{4}$	0.5160	0.3804	0.1035	0.5160	0.6195	3
$FM_{5}$	0.4335	0.4529	0.1136	0.4335	0.5471	9
$FM_{6}$	0.4418	0.4466	0.1116	0.4418	0.5534	8
$FM_{7}$	0.4927	0.3989	0.1083	0.4927	0.6010	5
$FM_{8}$	0.4634	0.4213	0.1152	0.4634	0.5786	6
$FM_{9}$	0.3853	0.4901	0.1246	0.3853	0.5099	10
$FM_{10}$	0.3859	0.4990	0.1151	0.3859	0.5010	11
$FM_{11}$	0.4476	0.4396	0.1127	0.4476	0.5603	7
$FM_{12}$	0.3090	0.5659	0.1251	0.3090	0.4351	12

**Table 13:** The comparison of final ranking in different methods.

Failure Mode	Method 1	Method 2	Method 3	Proposed Method
$FM_{1}$	2	2	2	2
$FM_{2}$	1	1	1	1
$FM_{3}$	5	6	6	4
$FM_{4}$	4	4	4	3
$FM_{5}$	9	11	9	9
$FM_{6}$	8	7	8	8
$FM_{7}$	3	5	3	5
$FM_{8}$	6	3	5	6
$FM_{9}$	10	9	11	10
$FM_{10}$	11	10	10	11
$FM_{11}$	7	8	7	7
$FM_{12}$	12	12	12	12
